# The national and provincial burden of medically attended influenza‐associated influenza‐like illness and severe acute respiratory illness in the Democratic Republic of Congo, 2013‐2015

**DOI:** 10.1111/irv.12601

**Published:** 2018-09-06

**Authors:** Pélagie Babakazo, Léopold Lubula, Wally Disasuani, Léonie Kitoko Manya, Edith Nkwembe, Naomi Mitongo, Hugo Kavunga‐Membo, Jean‐Claude Changachanga, Saleh Muhemedi, Benoit Kebela Ilunga, Emile Okitolonda Wemakoy, Jean‐Jacques Muyembe Tamfum, Joelle Kabamba‐Tshilobo, Stefano Tempia

**Affiliations:** ^1^ Kinshasa School of Public Health University of Kinshasa Kinshasa Democratic Republic of Congo; ^2^ Division de Lutte Contre la Maladie Ministry of Health Kinshasa Democratic Republic of Congo; ^3^ Institut National de Recherche Biomédicale Ministry of Health Kinshasa Democratic Republic of Congo; ^4^ Influenza and Monkeypox Program Centers for Disease Control and Prevention Kinshasa Democratic Republic of Congo; ^5^ Influenza Division Centers for Disease Control and Prevention Atlanta Georgia; ^6^ Influenza Program Centers for Disease Control and Prevention Pretoria South Africa; ^7^ Centre for Respiratory Diseases and Meningitis National Institute for Communicable Diseases of the National Health Laboratory Service Johannesburg South Africa; ^8^ MassGenics Duluth Georgia

**Keywords:** burden, Democratic Republic of Congo, influenza, influenza‐like illness, severe acute respiratory illness

## Abstract

**Background:**

Estimates of influenza‐associated outpatient consultations and hospitalizations are severely limited in low‐ and middle‐income countries, especially in Africa.

**Methods:**

We conducted active prospective surveillance for influenza‐like illness (ILI) and severe acute respiratory illness (SARI) at 5 healthcare facilities situated in Kinshasa Province during 2013‐2015. We tested upper respiratory tract samples for influenza viruses using a reverse transcription‐polymerase chain reaction assay. We estimated age‐specific numbers and rates of influenza‐associated ILI outpatient consultations and SARI hospitalizations for Kinshasa Province using a combination of administrative and influenza surveillance data. These estimates were extrapolated to each of the remaining 10 provinces accounting for provincial differences in prevalence of risk factors for pneumonia and healthcare‐seeking behavior. Rates were reported per 100 000 population.

**Results:**

During 2013‐2015, the mean annual national number of influenza‐associated ILI outpatient consultations was 1 003 212 (95% Confidence Incidence [CI]: 719 335‐1 338 050 ‐ Rate: 1205.3; 95% CI: 864.2‐1607.5); 199 839 (95% CI: 153 563‐254 759 ‐ Rate: 1464.0; 95% CI: 1125.0‐1866.3) among children aged <5 years and 803 374 (95% CI: 567 772‐1 083 291 ‐ Rate: 1154.5; 95% CI: 813.1‐1556.8) among individuals aged ≥5 years. The mean annual national number of influenza‐associated SARI hospitalizations was 40 361 (95% CI: 24 014‐60 514 ‐ Rate: 48.5; 95% CI: 28.9‐72.7); 25 452 (95% CI: 19 146‐32 944 ‐ Rate: 186.5; 95% CI: 140.3‐241.3) among children aged <5 years and 14 909 (95% CI: 4868‐27 570 ‐ Rate: 21.4; 95% CI: 28.9‐72.7) among individuals aged ≥5 years.

**Conclusions:**

The burden of influenza‐associated ILI outpatient consultations and SARI hospitalizations was substantial and was highest among hospitalized children aged <5 years.

## 1 INTRODUCTION

Influenza virus infections cause substantial morbidity and mortality globally, in particular among young children and older adults.^1^, ^2^, ^3^ In addition, global studies highlighted a higher burden of influenza‐associated mortality in Africa compared with other Regions.^1^ An elevated burden of influenza‐associated hospitalization among African children has also been reported.^2^, ^3^ However, the majority of influenza disease burden estimates available for global studies are from industrialized countries.

The World Health Organization (WHO) highlighted that there is a need for influenza disease burden estimates especially from low‐ and middle‐income countries.^4^ Such estimates would enable governments to make informed and evidence‐based decisions when allocating scarce resources and planning intervention strategies to limit the impact and spread of the disease. In addition, national estimates would assist to refine the global understanding of the burden of influenza‐associated illness and inform global public health priorities.

In recent years, influenza sentinel surveillance has been established in several African countries 5 and influenza virus infection has been found to be associated with mild and severe illness including death.^5^, ^6^ Nonetheless, national estimates of influenza‐associated hospitalization 7, 8, 9, 10, 11, 12 and outpatient consultations 7, 10, 13 across age groups remain limited in Africa. In the Democratic Republic of Congo (DRC), there are currently no recommendations for influenza immunization or treatment.

In this study, we aimed to estimate the national and provincial number and rates of medically attended influenza‐associated influenza‐like illness (ILI) outpatient consultation, and severe acute respiratory illness (SARI) hospitalization among persons of different age groups, DRC from January 2013 through December 2015.

## 2 METHODS

### 2.1 Data sources

#### 2.1.1 Data source 1: Number of respiratory hospitalizations and outpatient consultations in Kinshasa Province

We obtained the number of respiratory outpatient consultations and hospitalizations in Kinshasa Province by year and healthcare facility during January 2013 to December 2015 from the DRC Ministry of Health (MoH‐DRC) (Dr. Léopold Lubula personal communication), which collects aggregated and deidentified data on the number of outpatient consultations and admissions by syndrome from all healthcare facilities in Kinshasa Province.

#### 2.1.2 Data source 2: Retrospective record review of respiratory admissions and outpatient consultations in selected healthcare facilities

To assess the completeness of the administrative data reported to the MoH‐DRC (data source 1), we implemented an anonymized retrospective record review (using healthcare facilities consultation or admission books) of any respiratory outpatient consultation or admission in 5% of randomly selected healthcare facilities situated in Kinshasa Province from January 2013 through December 2015. We implemented a presurvey to compile a list of the most common respiratory outpatient consultations or admission diagnoses recorded by the attending clinicians in consultation or admission books. Respiratory outpatient consultations were considered any upper respiratory tract infections such as rhinitis, pharyngitis, or laryngitis; whereas respiratory admissions were considered any lower respiratory tract infections such as bronchitis, bronchiolitis, or pneumonia.

The sampling frame consisted of all public healthcare facilities (842) providing medical care in Kinshasa Province, including 584 (65%) primary health clinics where only outpatient medical care is provided, 235 (28%) policlinics where outpatient and limited inpatient medical care is provided, and 59 (7%) primary, secondary or tertiary hospitals or medical centers where inpatient and outpatient medical care is provided. Forty‐two healthcare facilities were randomly selected for the study, including 26/584 primary health clinics, 12/235 policlinics, and 4/59 hospitals or medical centers. The healthcare facilities implementing influenza virus surveillance described in Data source 3 were included in the sampling frame, but they were not selected using random selection procedures. For each identified respiratory outpatient consultation or admission gender, age, location of residence, consultation/admission diagnosis, and date and place of consultation/admission were recorded.

#### 2.1.3 Data source 3: Influenza virus surveillance among patients with ILI or SARI

We conducted active, prospective surveillance among outpatients with ILI at 2 clinics (Boyambi and RVA) and at the outpatient departments of 3 hospitals (Hôpital Général de Kinshasa, Hôpital Pédiatrique de Kalembelembe and Centre Hospitalier de Kingasani) situated in Kinshasa Province from January 2013 through December 2015. In addition, we conducted active, prospective hospital‐based surveillance among inpatients with SARI at the medical adult and pediatric wards of the 3 above mentioned hospitals during the same period.

A case of ILI was defined as an outpatient of any age presenting with a recorded temperature ≥38°C and cough or sore throat of duration of ≤7 days. A case of SARI was defined as a hospitalized person who had illness onset within 7 days of admission and who met age‐specific clinical inclusion criteria. A SARI case in children aged 2 days to <5 years included any hospitalized patient with cough or difficulty breathing and at least one of the following danger signs: unable to drink or breastfeed, lethargic, vomits everything, convulsion, chest in drawing, or stridor in a calm child. A SARI case in persons aged ≥5 years included any hospitalized patient with fever (≥38°C), cough, and shortness of breath or difficulty breathing.

The procedures of this surveillance program have been previously described.^14^ Briefly, trained surveillance staff (doctors, nurses, or laboratory technicians) completed case report forms that included demographic, clinical, and epidemiological information for all enrolled ILI and SARI cases. All respiratory admission or outpatient consultations and those meeting the ILI and SARI case definition were also recorded. In addition, respiratory specimens (nasopharyngeal and oropharyngeal swabs) were collected from all enrolled patients, placed in the same vial containing universal transport medium, stored at 4‐8°C, and transported to the national influenza laboratory (the Institut National de Recherche Biomédicale, Kinshasa, DRC) within 72 hours of collection for testing. Specimens were tested for influenza A and B viruses using a real‐time reverse transcription‐polymerase chain reaction assay.^14^ Influenza A‐positive samples were further subtyped.^15^ Verbal informed consent was obtained from all patients prior to data and specimen collection. For children aged <15 years, verbal consent was obtained from a parent or legal guardian.

#### 2.1.4 Data source 4: Prevalence of risk factors for pneumonia and healthcare‐seeking behavior for acute respiratory infection

We obtained the provincial‐level prevalence of known risk factors for pneumonia and the provincial data on healthcare‐seeking behavior among cases with acute respiratory infection (ARI) from the 2013‐2014 DRC Demographic and Health Survey (DHS).^16^

#### 2.1.5 Data source 5: Population denominators

Provincial age‐ and year‐specific mid‐year population denominators were obtained from projections of 1984 census data for DRC.^17^ DRC had an estimated population of 86 032 976 individuals [11 575 923 (13.5%) in Kinshasa Province] in 2015 of which 14 109 408 (16.4%) were children aged <5 years.

### 2.2 Estimation of the national number and rate of SARI and influenza‐associated SARI hospitalizations

To estimate the national number and rates of SARI and influenza‐associated SARI hospitalization, we used a four‐step approach. In Step 1, we estimated the SARI hospitalization rate in Kinshasa Province (considered to be the base province in our estimation approach). In Step 2, we estimated the SARI hospitalization rates for the other provinces from the base province using a previously described methodology.^8^, ^11^, ^12^, ^18^, ^19^ In Step 3, we estimated the influenza‐associated SARI hospitalization rate using available virological surveillance data for influenza. In Step 4, we obtained the number of SARI and influenza‐associated SARI hospitalizations using the estimated rates and the population at risk in each province.

**Figure 1:**
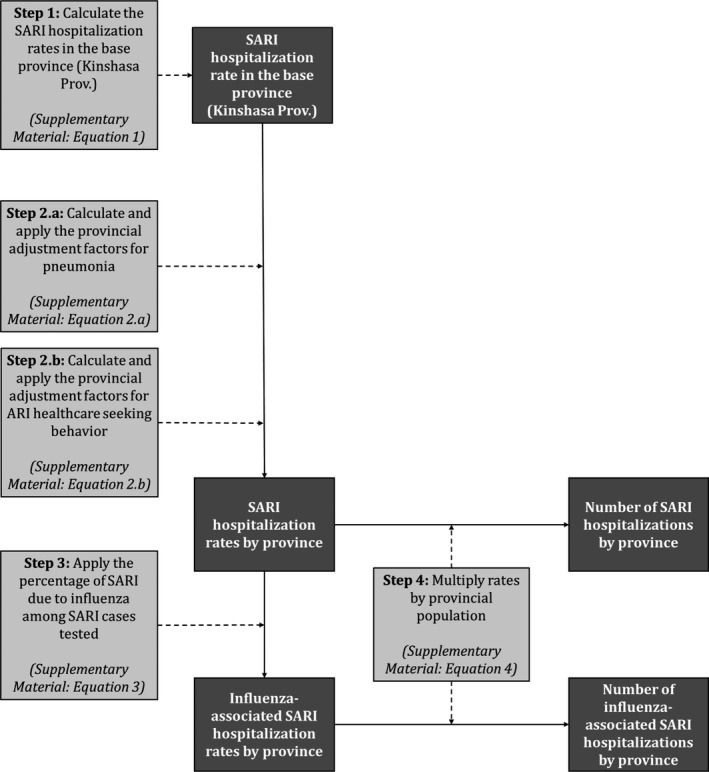
Method used to estimate the numbers and rates of severe acute respiratory illness (SARI) and influenza‐associated SARI hospitalizations in the Democratic Republic of Congo, 2013‐2015. Data inputs steps are in light gray boxes, and data outputs are in dark gray boxes

The description of the estimation approach for each step is provided below and in Figure 1. All estimates were obtained overall and within the following age categories: <1, 1‐4, 5‐24, 25‐44, 45‐64, ≥65, <5, and ≥5 years of age. Rates were reported per 100 000 population. We reported mean annual estimates over the study period.

#### 2.2.1 Step 1: Estimation of the SARI hospitalization rate in Kinshasa Province

To estimate the SARI hospitalization rate in Kinshasa Province, we followed WHO guidelines for estimating the disease burden associated with seasonal influenza.^4^ First we estimated the number of any respiratory hospitalization in Kinshasa Province by adjusting the number of respiratory hospitalizations reported to the MoH‐DRC (data source 1) by the estimated proportion of underreporting. The proportion of underreporting was obtained by comparing the number of respiratory admissions reported to the MoH‐DRC (data source 1) from the healthcare facilities where the retrospective record review was implemented and those identified from the record review (data source 2). Thereafter, we applied the proportion of inpatients with respiratory illness that met the SARI case definition from the hospitals where SARI surveillance was implemented (data source 3). Lastly, we obtained the SARI hospitalization rate for Kinshasa Province by dividing the total estimated number of SARI hospitalizations by the mid‐year population estimate (data source 5).

#### 2.2.2 Step 2: Estimation of SARI hospitalization rates in other provinces

Estimates of SARI hospitalization rates for the other 10 provinces in DRC were derived by adjusting the Kinshasa Province rate (base province—obtained in Sep 1) for the provincial‐level prevalence of known risk factors for pneumonia obtained from the DHS (data source 4) as previously described (Step 2.a).^8^, ^11^, ^12^, ^18^, ^19^ Risk factors included HIV infection, exposure to indoor air pollution, and crowding for all ages, and, in addition, for children aged <5 years malnutrition, low birthweight and nonexclusive breastfeeding.^8^, ^11^, ^12^, ^18^, ^19^ The relative risk of SARI associated with each risk factor was determined from the published literature.^8^, ^11^, ^12^, ^18^, ^19^, ^20^, ^21^, ^22^ In addition, we adjusted the provincial rates by the proportion of ARI cases seeking care in the given province to the proportion of ARI cases seeking care in the base province using data from the DHS (data source 4) as previously described (Step 2.b).^8^, ^11^, ^12^, ^18^, ^19^ We used the healthcare‐seeking behavior among ARI cases as a proxy for SARI cases. An adjustment factor >1 resulted in a greater SARI hospitalization rate in the given province relative to the base province and vice versa. The equations used for the provincial adjustments and the estimated adjustment factors (Table S1) are provided in the Supplementary Material.

#### 2.2.3 Step 3: Estimation of influenza‐associated SARI hospitalization rates in all provinces

We estimated the provincial rates of influenza‐associated SARI hospitalization by multiplying the estimated provincial SARI hospitalization rates (obtained in Step 1 and 2) by the influenza virus detection rate obtained from influenza sentinel surveillance implemented among inpatients with SARI (data source 3).^8^, ^11^, ^12^, ^18^, ^19^

#### 2.2.4 Step 4: Estimation of the number of SARI and influenza‐associated SARI hospitalizations in all provinces

We estimated the provincial number of SARI and influenza‐associated SARI hospitalizations by multiplying the provincial SARI (obtained in Steps 1 and 2) and influenza‐associated SARI (obtained in Step 3) hospitalization rates by the population at risk in each province over the study period.^8^, ^11^, ^12^, ^18^, ^19^

### 2.3 Estimation of the national number and rate of ILI and influenza‐associated ILI outpatient consultations

**Figure 2:**
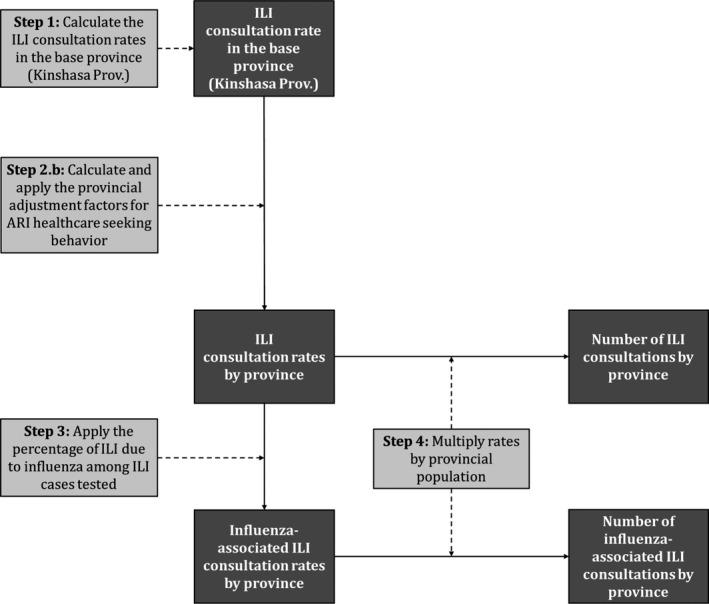
Method used to estimate the numbers and rates of influenza‐like illness (ILI) and influenza‐associated ILI outpatient consultations in the Democratic Republic of Congo, 2013‐2015. Data inputs steps are in light gray boxes, and data outputs are in dark gray boxes

To estimate the national number and rates of ILI and influenza‐associated ILI outpatient consultation, we used the same approach as for SARI with the exception that for the provincial adjustment, we used only the healthcare‐seeking behavior for ARI in the given province to the base province and we used the influenza virus detection rate obtained from influenza sentinel surveillance implemented among outpatients with ILI (data source 3) (Figure 2).

### 2.4 Calculation of confidence intervals

We obtained the 95% confidence intervals (CI) using bootstrap resampling over 1000 replications for all parameters included in the calculations.^8^, ^11^, ^12^, ^18^, ^19^ This included (a) the age‐, year‐, and healthcare facility‐specific proportion of underreporting (data source 1 and 2), (b) the age‐, year‐, and healthcare facility‐specific proportion of ILI/SARI cases over total respiratory consultations/admissions (data source 3), (c) the provincial prevalence of the risk factors for pneumonia (data source 4), (d) the provincial proportion of ARI cases seeking care (data source 4), and (e) the age‐ and year‐specific influenza virus percentage positive among ILI/SARI cases tested (data source 3). The lower and upper limits of the 95% CI were the 2.5th and 97.5th percentiles of the estimated values obtained from the 1000 resampled datasets, respectively.^8^, ^11^, ^12^, ^18^, ^19^ The statistical analysis was implemented using Stata 14.2 (StataCorp, College Station, Texas, USA).

### 2.5 Ethical approval

The influenza sentinel surveillance (data source 3) and the collection of aggregated data on any respiratory consultations/admissions (data source 2) were deemed nonresearch by the MoH‐DRC and the US Centers for Disease Control and Prevention. The number of respiratory consultations/admissions in Kinshasa Province reported to the MoH‐DRC (data source 1), the DHS (data source 4) and the census data (data source 5) were publicly available.

## 3 RESULTS

### 3.1 Reported number and retrospective record review of respiratory illness in selected healthcare facilities in Kinshasa Province

During 2013‐2015, there were 39 654 respiratory illnesses recorded from the retrospective record review at the 42 randomly selected healthcare facilities (data source 2). Of these, 35 628 (89.8%) were outpatient consultations and 4026 (10.2%) were hospitalizations, of which 20 679 (58.0%) and 3112 (77.3%) were reported to the MoH‐DRC (data source 1). Children aged <5 years accounted for 47.2% (16 830/35 628) of outpatient consultations and 73.2% (2948/4026) of hospitalizations associated with respiratory illness (data source 2).

### 3.2 Influenza virus surveillance among patients with ILI or SARI

**Figure 3:**
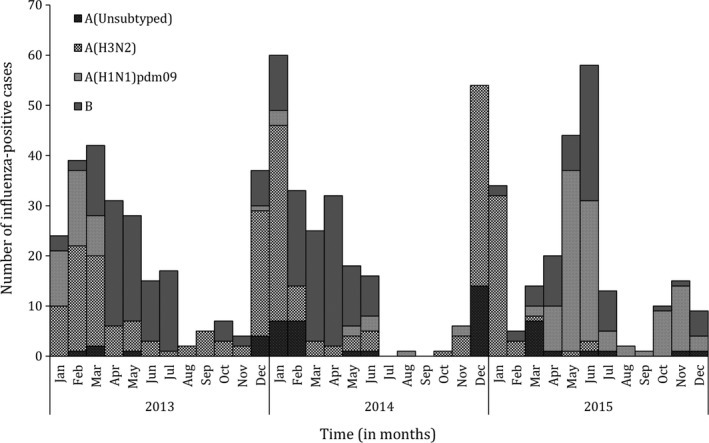
Monthly number of influenza‐positive specimens among patients with influenza‐like illness or severe acute respiratory illness from 5 surveillance sites in Kinshasa Province, Democratic Republic of Congo, 2013‐2015

During the study period, we enrolled and tested 7467 patients, of which 4297 (57.5%) had ILI and 3170 (42.5%) had SARI. Influenza viruses were detected in 9.7% (722/7467) of specimens. Of these, 244 (33.8%) were influenza A(H3N2), 153 (21.2%) were influenza A(H1N1)pdm09, 50 (6.9%) were influenza A not subtyped, and 275 (38.1%) were influenza B viruses (Figure 3). In all age groups among patients with ILI, influenza viruses were detected in 10.7% (462/4297) of specimens; in 12.3% (256/2074) and 9.3% (206/2223) of specimens among individuals aged <5 and ≥5 years, respectively. In all age groups among patients with SARI, influenza viruses were detected in 8.2% (260/3170) of specimens; in 9.4% (208/2208) and 5.4% (52/962) of specimens among individuals aged <5 and ≥5 years, respectively.

### 3.3 National number and rate of ILI and influenza‐associated ILI outpatient consultations

The estimated mean annual number of ILI outpatient consultations was 9 896 684 (rate: 11 890.0 per 100 000 population); 1 586 818 (16.0%) (rate: 11 624.5 per 100 000 population) and 8 309 866 (84.0%%) (rate: 11 942.1 per 100 000 population) among individuals aged <5 and ≥5 years, respectively (Table 1). The estimated mean annual rate of ILI outpatient consultations was highest among individuals aged 25‐44 years (13 529.6 per 100 000 population) and lowest among individuals aged ≥65 years (6340.0 per 100 000 population).

**Table 1:** Estimated mean annual numbers and rates of influenza‐like illness and influenza‐associated influenza‐like illness outpatient consultations, Democratic Republic of Congo, 2013‐2015

Age group^a^ (in y)	ILI outpatient consultations		Influenza‐associated ILI outpatient consultations	
	Number (95% CI)	Rate (95% CI)^b^	Number (95% CI)	Rate (95% CI)^b^
<1	309 110 (263 266‐362 206)	11 253.6 (9584.6‐13 186.6)	33 749 (23 567‐45 448)	1228.7 (858.0‐1654.6)
1‐4	1 277 708 (1 085 757‐1 497 510)	11 718 (9957.6‐13 733.8)	166 090 (129 997‐209 312)	1523.2 (1192.2‐1919.6)
5‐24	4 759 941 (4 065 403‐5 597 268)	12 271.8 (10 481.2‐14 430.5)	629 573 (46 2951‐828 539)	1623.1 (1193.6‐2136.1)
25‐44	2 421 202 (2 061 756‐2 823 498)	13 529.6 (11 521.0‐15 777.6)	110 390 (68 648‐157 246)	616.9 (383.6‐878.7)
45‐64	907 083 (773 611‐1 066 884)	9644.1 (8225.0‐11 343.1)	51 539 (30 516‐74 858)	548.0 (324.4‐795.9)
≥65	221 640 (188 487‐260 973)	6340.0 (5391.7‐7465.1)	11 872 (3658‐22 649)	339.6 (104.6‐647.9)
<5	1 586 818 (1 349 023‐1 859 716)	11 624.5 (9882.5‐13 623.7)	199 839 (153 563‐254 759)	1464.0 (1125.0‐1866.3)
≥5	8 309 866 (7 089 256‐9 748 623)	11 942.1 (10 187.9‐14 009.7)	803 374 (565 772‐1 083 291)	1154.5 (813.1‐1556.8)
All	9 896 684 (8 438 278‐11 608 338)	11 890.0 (10 137.9‐13 946.4)	1 003 213 (719 335‐1 338 050)	1205.3 (864.2‐1607.5)
Province^c^				
Kinshasa	1 455 563 (1 452 614‐1 458 617)	13 156.3 (13 129.7‐13 183.9)	147 549 (111 474‐185 755)	1333.6 (1007.6‐1679)
Bas‐Congo	550 701 (431 433‐683 879)	10 208.0 (7997.2‐12 676.6)	55 825 (38 516‐76 316)	1034.8 (713.9‐1414.6)
Bandundu	979 977 (767 573‐1 210 840)	10 625.2 (8322.3‐13 128.3)	99 339 (68 577‐138 475)	1077.1 (743.5‐1501.4)
Équateur	888 056 (725 129‐1 101 709)	10 625.2 (8675.8‐13 181.4)	90 021 (63 128‐122 511)	1077.1 (755.3‐1465.8)
Orientale	812 217 (693 386‐960 188)	9234.5 (7883.4‐10 916.8)	82 334 (59 547‐108 908)	936.1 (677.0‐1238.2)
Nord‐Kivu	536 171 (407 044‐682 561)	8316.6 (6313.7‐10 587.3)	54 350 (36 457‐75 453)	843.0 (565.5‐1170.4)
Sud‐Kivu	657 881 (507 210‐828 836)	11 765.6 (9071.0‐14 823.0)	66 688 (45 411‐93 272)	1192.7 (812.1‐1668.1)
Maniema	342 687 (289 968‐406 576)	15 159 (12 826.9‐17 985.2)	34 738 (24 859‐46 370)	1536.7 (1099.6‐2051.2)
Katanga	1 864 705 (1 640 879‐2 136 163)	14 658.3 (12 898.9‐16 792.3)	189 022 (139 343‐247 363)	1485.9 (1095.4‐1944.5)
Kasaï Oriental	1 153 151 (959 733‐1 369 800)	15 492.8 (12 894.2‐18 403.5)	116 892 (83 350‐156 013)	1570.5 (1119.8‐2096.1)
Kasaï Occidental	655 575 (563 312‐769,172)	11 042.4 (9488.4‐12 955.8)	66 455 (48 675‐87 616)	1119.4 (819.9‐1475.8)

ILI, influenza‐like illness; CI, confidence intervals.

a National estimates by age group.

b Rates expressed per 100 000 population.

c Provincial estimates for all age groups.

The estimated mean annual number of influenza‐associated ILI outpatient consultations was 1 003 213 (rate: 1205.3 per 100 000 population); 199 839 (19.9%) (rate: 1464 per 100 000 population) and 803 374 (80.1%) (rate: 1154.5 per 100 000 population) among individuals aged <5 and ≥5 years, respectively (Table 1). The estimated mean annual rate of influenza‐associated ILI outpatient consultations was highest among individuals aged 5‐24 years (1623.1 per 100 000 population) and lowest among individuals aged ≥65 years (339.6 per 100 000 population).

**Figure 4:**
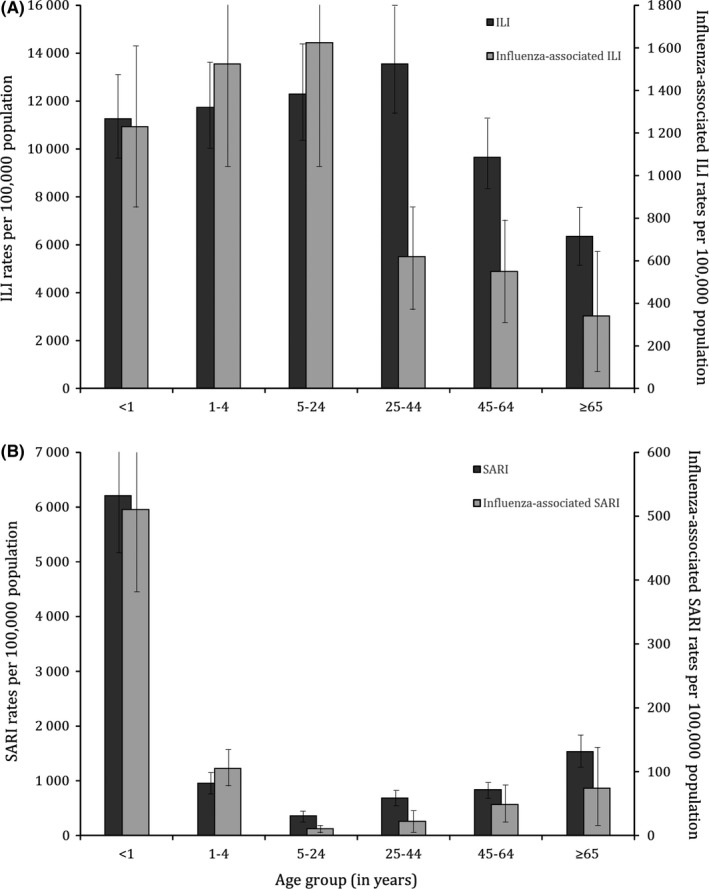
Mean annual estimates of mild or severe respiratory illness and influenza‐associated mild or severe respiratory illness rates by age group, Democratic Republic of Congo, 2013‐2015. A, Influenza‐like illness (ILI); B, Severe acute respiratory illness (SARI)

An inverted U‐shaped trend of the magnitude of the ILI and influenza‐associated ILI outpatient consultation rates was observed across age groups (Table 1 and Figure 4 panel A). No substantial differences (with overlapping CIs) of the ILI and influenza‐associated ILI outpatient consultations rates were observed across provinces (Table 1). The provincial number and rates of ILI and influenza‐associated ILI outpatient consultations by age group are provided in Table S2.

### 3.4 National number and rate of SARI and influenza‐associated SARI hospitalizations

The estimated mean annual number of SARI hospitalizations was 661 911 (rate: 795.2 per 100 000 population); 273 321 (41.3%) (rate: 2002.3 per 100 000 population) and 388 590 (58.7%) (rate: 558.4 per 100 000 population) among individuals aged <5 and ≥5 years, respectively (Table 2). The estimated mean annual rate of SARI hospitalization was highest among children aged <1 year (6204.3 per 100 000 population) and lowest among individuals aged 5‐24 years (348.5 per 100 000 population).

**Table 2:** Estimated mean annual numbers and rates of severe acute respiratory illness and influenza‐associated severe acute respiratory illness hospitalizations, Democratic Republic of Congo, 2013‐2015

Age group^a^ (in y)	SARI hospitalizations		Influenza‐associated SARI hospitalizations	
	Number (95% CI)	Rate (95% CI)^b^	Number (95% CI)	Rate (95% CI)^b^
<1	170 418 (143 545‐202 407)	6204.3 (5225.9‐7368.9)	14 001 (10 540‐17 962)	509.7 (383.7‐653.9)
1‐4	102 903 (86 508‐122 012)	943.7 (793.4‐1119.0)	11 451 (8607‐14 982)	105.0 (78.9‐137.4)
5‐24	135 177 (115 308‐159 159)	348.5 (297.3‐410.3)	3918 (1189‐7492)	10.1 (3.1‐19.3)
25‐44	122 265 (103 999‐143 012)	683.2 (581.1‐799.1)	3925 (1129‐7331)	21.9 (6.3‐41.0)
45‐64	77 724 (65 983‐91 533)	826.4 (701.5‐973.2)	4492 (1961‐7449)	47.8 (20.8‐79.2)
≥65	53 424 (45 412‐62 969)	1528.2 (1299.0‐1801.2)	2574 (590‐5299)	73.6 (16.9‐151.6)
<5	273 321 (230 052‐324 419)	2002.3 (1685.3‐2376.6)	25 452 (19 146‐32 944)	186.5 (140.3‐241.3)
≥5	388 590 (330 702‐456 672)	558.4 (475.3‐656.3)	14 909 (4868‐27 570)	21.4 (7.0‐39.6)
All	661 911 (560 754‐781 091)	795.2 (673.7‐938.4)	40 361 (24 014‐60 514)	48.5 (28.9‐72.7)
Province^c^				
Kinshasa	93 404 (92 606‐94 230)	844.2 (837.0‐851.7)	5605 (3515‐8041)	50.7 (31.8‐72.7)
Bas‐Congo	35 742 (27 905‐44 634)	662.5 (517.3‐827.4)	2217 (1291‐3340)	41.1 (23.9‐61.9)
Bandundu	62 616 (48 791‐77 974)	678.9 (529.0‐845.4)	3839 (2208‐5916)	41.6 (23.9‐64.1)
Équateur	57 892 (46 947‐71 214)	692.7 (561.7‐852.0)	3544 (2074‐5341)	42.4 (24.8‐63.9)
Orientale	56 027 (47 560‐66 441)	637.0 (540.7‐755.4)	3376 (2010‐5087)	38.4 (22.8‐57.8)
Nord‐Kivu	36 969 (28 161‐47 358)	573.4 (436.8‐734.6)	2257 (1281‐3522)	35.0 (19.9‐54.6)
Sud‐Kivu	41 579 (31 739‐52 810)	743.6 (567.6‐944.4)	2572 (1478‐3962)	46.0 (26.4‐70.9)
Maniema	25 134 (21 088‐30 192)	1111.8 (932.8‐1335.5)	1544 (917‐2326)	68.3 (40.6‐102.9)
Katanga	127 536 (111 694‐147 459)	1002.6 (878‐1159.2)	7731 (4680‐11 547)	60.8 (36.8‐90.8)
Kasaï Oriental	79 829 (65 902‐95 357)	1072.5 (885.4‐1281.1)	4884 (2873‐7303)	65.6 (38.6‐98.1)
Kasaï Occidental	45 183 (38 363‐53 425)	761.1 (646.2‐899.9)	2792 (1690‐4130)	47.0 (28.5‐69.6)

SARI, severe acute respiratory illness; CI, confidence intervals.

a National estimates by age group.

b Rates expressed per 100 000 population.

c Provincial estimates for all age groups.

The estimated mean annual number of influenza‐associated SARI hospitalization was 40 361 (rate: 48.5 per 100 000 population); 25 452 (63.1%) (rate: 186.5 per 100 000 population) and 14 909 (36.9%) (rate: 21.4 per 100 000 population) among individuals aged <5 and ≥5 years, respectively (Table 2). The estimated mean annual rate of influenza‐associated SARI hospitalization was highest among children aged <1 year (509.7 per 100 000 population) and lowest among individuals aged 5‐24 years (10.1 per 100 000 population).

A U‐shaped trend of the magnitude of the SARI and influenza‐associated SARI hospitalization rates was observed across age groups (Table 2 and Figure 4 panel B). No substantial differences (with overlapping CIs) of the SARI and influenza‐associated SARI hospitalizations rates were observed across provinces (Table 2). The provincial number and rates of SARI and influenza‐associated SARI hospitalizations by age group are provided in Table S3.

## 4 DISCUSSION

We reported national and provincial estimates of medically attended influenza‐associated ILI and SARI in DRC over a 3‐year period. Influenza virus infections were associated with mild (ILI) and severe (SARI) respiratory illness across age groups. However, the rates of influenza‐associated ILI outpatient consultations were highest among individuals aged 5‐24 years, whereas the highest rates of influenza‐associated SARI hospitalization were observed among individuals aged <5 and ≥65 years, with children aged <5 years accounting for 63.1% of all influenza‐associated SARI hospitalizations. Higher rates of influenza‐associated SARI hospitalizations among young children and older adults have also been reported in other studies.^7^, ^8^, ^9^, ^10^, ^11^, ^12^

Our estimated rates of influenza‐associated ILI outpatient consultation of 1205 per 100 000 population were generally consistent with those of other studies conducted in Africa: 895 per 100 000 population in Ghana,^7^ 720 per 100 000 population in Kenya 13 and 1337 per 100 000 population in South Africa.^10^ In our study, individuals aged 5‐24 years experienced the highest rates of influenza‐associated ILI outpatient consultation. This was similar to a study conducted in the United States whereby individuals aged 2‐17 years experienced the highest rates of influenza‐associated medically attended ILI,^23^ suggesting the importance of this age group in the transmission of influenza viruses.^24^

Estimates of influenza‐associated respiratory hospitalizations among African children aged <5 years obtained from a global study were 174 per 100 000 population.^3^ Studies conducted in Africa reported estimated rates (per 100 000 population) of influenza‐associated SARI hospitalization among children aged <5 years of 135 in Ghana,^7^ 100 in Kenya,^8^ 128 in Madagascar,^12^ 168 in Rwanda,^9^ 156 in South Africa,^10^ and 187 in Zambia.^11^ Our estimated rates of influenza‐associated SARI hospitalization among children aged <5 years of 186 per 100 000 population are generally consistent with the estimates obtained from studies conducted in Africa, although higher compared to those of other regions.^3^

In our study, the estimated rate of influenza‐associated SARI hospitalization among individuals aged ≥5 years (21 per 100 000 population) was almost 9 times lower than that estimated among children aged <5 years. Lower rates (per 100 000 population) of influenza‐associated SARI hospitalization among individuals aged ≥5 compared to <5 years were observed also in other studies conducted in Africa: 7 in Kenya,^8^ 12 in Madagascar,^12^ 11 in Rwanda,^9^ 31 in South Africa,^10^ and 13 in Zambia.^11^

In our study, we did not observe substantial differences (with overlapping CIs) in the provincial rates of medically attended influenza‐associated ILI or SARI. For SARI, this was observed also in studies conducted in Madagascar,^12^ Rwanda,^9^ South Africa,^19^ and Zambia.^11^ This may suggest that geographical variations within countries may not significantly affect influenza disease burden estimates.

Our study has limitations that warrant discussion. First, whereas we estimated national numbers and rates of medically attended influenza‐associated ILI or SARI using a previously described methodology,^8^, ^11^, ^12^, ^18^, ^19^ our estimates were derived from influenza surveillance conducted at selected healthcare facilities situated in Kinshasa Province. The influenza virus detection rate may vary in different locations in DRC; however, this could not be investigated. Nonetheless, the influenza virus detection rate among SARI cases was found to be similar across 15 African countries including DRC (median: 8.9%; interquartile range: 5.7%‐11.6%) 5 and our rates of medically attended influenza‐associated ILI or SARI were similar to those obtained in other African Countries.^7^, ^8^, ^9^, ^10^, ^11^ In addition, we used bootstraping for the calculation of the CIs to account for the level of uncertainty associated with all adjustments used in our estimation approach as previously described.^8^, ^11^, ^12^, ^18^, ^19^ Second, the coefficient used for the provincial adjustment accounts for the magnitude of the risk and the provincial difference in the prevalence of the selected risk factors individually, but it does not account for the proportion of individuals that are exposed to two or more risk factors simultaneously in each province. In addition, the interaction effect of multiple co‐occurring risk factors on increased risk of pneumonia is unknown. Third, we were unable to account for patients hospitalized for respiratory illness that did not meet the ILI or SARI case definitions because we implemented influenza sentinel surveillance only among individuals meeting these case definitions. This may lead to an underestimation of influenza‐associated disease burden. Last, ecological studies have suggested that influenza viruses are also responsible for hospitalizations and deaths among patients presenting with circulatory illnesses or even syndromes different than respiratory and circulatory 25, 26, 27, 28 which we did not account for in our estimates. In addition, individuals that may have developed influenza‐associated mild or severe illness, but did not seek care would have been missed in our study. A large proportion of nonmedically attended influenza‐associated ILI and SARI has been reported in other studies conducted in Africa^8^, ^13^, ^18^, ^19^; hence, our estimates should be considered minimum estimates. Cultural differences and differential access to healthcare across different countries can also play a role in differential healthcare‐seeking behavior that in return may be responsible for differences in outpatient consultation and hospitalization rates.

In conclusion, we estimated a large number of influenza‐associated ILI outpatient consultations and SARI hospitalizations in DRC. The hospitalization rates were highest in children aged <5 years and individuals aged ≥65 years. These estimates provide the foundation for future cost‐effectiveness studies to potentially guide influenza immunization policies. Should an influenza vaccination program be introduced in DRC, young children and the elderly may benefit most from annual influenza immunization. No influenza vaccine is licensed for children aged <6 months, but this group may be protected through the vaccination of their mothers during pregnancy.^29^, ^30^ Nonetheless, given the limited financial resources in our setting, estimation of the disease burden associated with other pathogens should also be considered to inform prioritization of interventions.

## CONFLICT OF INTEREST

All authors declare that they have no commercial or other associations that may pose a conflict of interest.

## AUTHOR CONTRIBUTIONS

Pélagie Babakazo, Léopold Lubula, Wally Disasuani, Léonie Kitoko Manya, Joelle Kabamba‐Tshilobo, and Stefano Tempia performed study concept and design. Pélagie Babakazo, Léopold Lubula, Wally Disasuani, Léonie Kitoko Manya, Edith Nkwembe, Naomi Mitongo, Hugo Kavunga‐Membo, Jean‐Claude Changachanga, Saleh Muhemedi, Joelle Kabamba‐Tshilobo, and Stefano Tempia carried out acquisition, analysis, or interpretation of data. Pélagie Babakazo and Stefano Tempia drafted the manuscript. Pélagie Babakazo, Léopold Lubula, Wally Disasuani, Léonie Kitoko Manya, Edith Nkwembe, Naomi Mitongo, Hugo Kavunga‐Membo, Jean‐Claude Changachanga, Saleh Muhemedi, Benoit Kebela Ilunga, Emile Okitolonda Wemakoy, Jean‐Jacques Muyembe Tamfum, Joelle Kabamba‐Tshilobo, and Stefano Tempia involved in critical revision of the manuscript for important intellectual content. All authors take responsibility for the integrity of the data and the accuracy of the data analysis.

## DISCLAIMER

The findings and conclusions in this report are those of the authors and do not necessarily represent the official position of the US Centers for Disease Control and Prevention, USA, or the DRC Ministry of Health.

## ETHICS

The influenza sentinel surveillance and the collection of aggregated data on any medical, respiratory, and SARI hospitalizations were deemed nonresearch by the DRC‐MoH and the US Centers for Disease Control and Prevention. The number of respiratory hospitalizations and outpatient consultations in Kinshasa Province, the DHS, and the census data were publicly available.
