# Supplementary material for: The national and provincial burden of medically attended influenza‐associated influenza‐like illness and severe acute respiratory illness in the Democratic Republic of Congo, 2013‐2015
Source: Influenza Other Respir Viruses. 2018 Sep 6;12(6):695–705. doi: 10.1111/irv.12601 (PMC6185889; doi:10.1111/irv.12601)
Supplement: Supplementary file 1 [file IRV-12-695-s001.docx]

**The National and Provincial Burden of Medically-Attended Influenza-Associated Influenza-Like-Illness and Severe Acute Respiratory Illness in the Democratic Republic of Congo, 2013-2015 (Supplementary Material)**

**Authors**

Pélagie Babakazo^1^, Léopold Lubula^2^, Wally Disasuani^1^, Léonie Kitoko Manya^2^, Edith Nkwembe^3^, Naomi Mitongo^3^, Hugo Kavunga-Membo^3^, Jean-Claude Changachanga^3^, Saleh Muhemedi^1^, Benoit Kebela Ilunga^2^, Emile Okitolonda Wemakoy^1^, Jean-Jacques Muyembe Tamfum^3^, Joelle Kabamba-Tshilobo^4^, Stefano Tempia^5,6,7,8^

**Affiliations**

^1^Kinshasa School of Public Health, University of Kinshasa, Kinshasa, Democratic Republic of Congo.

^2^Division de Lutte Contre la Maladie, Ministry of Health, Kinshasa, Democratic Republic of Congo.

^3^Institut National de Recherche Biomédicale, Ministry of Health, Kinshasa, Democratic Republic of Congo.

^4^Influenza and Monkeypox Program, Centers for Disease Control and Prevention, Kinshasa, Democratic Republic of Congo.

^5^Influenza Division, Centers for Disease Control and Prevention, Atlanta, Georgia, USA.

^6^Influenza Program, Centers for Disease Control and Prevention, Pretoria, South Africa.

^7^Centre for Respiratory Diseases and Meningitis, National Institute for Communicable Diseases of the National Health Laboratory Service, Johannesburg, South Africa.

^8^MassGenics, Duluth, Georgia, USA.

**METHODS**

**Equations used to estimate the provincial numbers and rates of SARI and influenza-associated SARI hospitalizations**

***Equation 1:*** *SARI hospitalization rates in the base province (Kinshasa Province)*

$${RS}_{H,B}=\frac{{SARI}_{H,B}}{{Pop}_{B}}$$

Where:

${RS}_{H,B}$ = Base rate of hospitalized SARI

${SARI}_{H,B}$ = Number of total cases meeting SARI case definition hospitalized at in Kinshasa Province

${Pop}_{B}$ = Population of Kinshasa Province

***Equation 2.a:*** *Calculation of adjustment for risk factors at the provincial level for base SARI hospitalization rates*

$${Adj}_{Y}=\left( 1+\sum_{i} \left( P_{i,Y}-P_{i,B} \right)\times\left( {RR}_{i}-1 \right) \right)$$

Where:

${Adj}_{Y}$ = Adjustment factor for province Y for risk factors of SARI

$P_{i,Y}$ = Prevalence of risk factor *i* in province Y

$P_{i,B}$= Prevalence of risk factor *i* in base province

${RR}_{i}$ = Relative risk of SARI due to risk factor *i*

***Equation 2.b:*** *SARI hospitalization rates in the given province after adjustment for risk factors and healthcare-seeking behavior*

$${RS}_{H,Y}={RS}_{H,B} \times{Adj}_{Y} \times\frac{{DHS}_{H,Y}}{{DHS}_{H,B}}$$

Where:

${RS}_{H,Y}$ = Rate of hospitalized SARI in province Y

${DHS}_{H,Y}$ = Proportion of ARI cases seeking care in province Y (from DHS)

${DHS}_{H,B}$ = Proportion of ARI cases seeking care in base province (from DHS)

***Equation 3:*** *Influenza-associated SARI hospitalization rates in all province*

$${RI}_{H,Y}={RS}_{H,Y} \times I$$

Where:

${RI}_{H,Y}$= Rate of hospitalized influenza-associated SARI in province Y

(including base province)

$I$ = Proportion of hospitalized SARI cases testing positive for influenza

***Equation 4:*** *Number of SARI and influenza-associated SARI hospitalizations in all provinces*

$${NS}_{H,Y}={RS}_{H,Y} \times P{op}_{Y}$$

Where:

${NS}_{H,Y}$= Number of hospitalized SARI cases in province Y (including base

province)

$P{op}_{Y}$= Population in province Y (including base province)

$${NI}_{H,Y}={RI}_{H,Y} \times P{op}_{Y}$$

Where:

${NI}_{H,Y}$= Number of hospitalized influenza-associated SARI cases in province Y

(including base province)

$P{op}_{Y}$= Population in province Y (including base province)

**RESULTS**

**Table S1: Adjustment factors for provincial estimates of severe acute respiratory illness hospitalization rates, Democratic Republic of Congo, 2013-2015**

| **Age group (in years)** | **Adjustment factors** | | |
| --- | --- | --- | --- |
|  | **Risk factors for pneumonia** | **ARI healthcare seeking behavior^a^** | **Combined** |
| **Kinshasa (Base province)** | | | |
| <5 | 1 | 1 | 1 |
| ≥5 | 1 | 1 | 1 |
| **Bas-Congo** | | | |
| <5 | 1.0831 | 0.7759 | 0.8762 |
| ≥5 | 0.9460 | 0.7759 | 0.7340 |
| **Bandundu** | | | |
| <5 | 1.0352 | 0.8076 | 0.8360 |
| ≥5 | 0.9526 | 0.8076 | 0.7693 |
| **Équateur** | | | |
| <5 | 1.0572 | 0.8076 | 0.8538 |
| ≥5 | 0.9756 | 0.8076 | 0.7879 |
| **Orientale** | | | |
| <5 | 1.0990 | 0.7019 | 0.7713 |
| ≥5 | 1.0686 | 0.7019 | 0.7500 |
| **Nord-Kivu** | | | |
| <5 | 1.1152 | 0.6321 | 0.7049 |
| ≥5 | 1.0386 | 0.6321 | 0.6565 |
| **Sud-Kivu** | | | |
| <5 | 1.0494 | 0.8942 | 0.9384 |
| ≥5 | 0.9268 | 0.8942 | 0.8288 |
| **Maniema** | | | |
| <5 | 1.2581 | 1.1522 | 1.4496 |
| ≥5 | 1.100 | 1.1522 | 1.2674 |
| **Katanga** | | | |
| <5 | 1.0945 | 1.1141 | 1.2194 |
| ≥5 | 1.0458 | 1.1141 | 1.1651 |
| **Kasaï Oriental** | | | |
| <5 | 1.1412 | 1.1776 | 1.3439 |
| ≥5 | 1.0408 | 1.1776 | 1.2256 |
| **Kasaï Occidental** | | | |
| <5 | 1.1469 | 0.8393 | 0.9626 |
| ≥5 | 1.0132 | 0.8393 | 0.8504 |

Abbreviations: ARI: acute respiratory infection.

^a^ Adjustment factors for ARI used also for provincial adjustments of influenza-like illness.

**Table S2: Estimated mean annual numbers and rates of influenza-like illness and influenza-associated influenza-like illness outpatient consultations by province and age group, Democratic Republic of Congo, 2013-2015**

| **Age group (in years)** | **ILI outpatient consultations** | | **Influenza-associated ILI outpatient consultations** | |
| --- | --- | --- | --- | --- |
|  | **Number (95% CI)** | **Rate (95% CI)^a^** | **Number (95% CI)** | **Rate (95% CI) ^a^** |
| **Kinshasa** | | | | |
| <1 | 45,463 (45,248-45,696) | 12,452.2 (12,393.2-12,516.1) | 4,964 (3,612-6,284) | 1,359.5 (989.3-1,721.2) |
| 1-4 | 187,920 (187,410-188,441) | 12,966.0 (12,930.8-13,001.9) | 24,428 (20,553-28,618) | 1,685.4 (1,418.1-1,974.6) |
| 5-24 | 700,072 (699,158-701,052) | 13,578.8 (13,561-13,597.8) | 92,595 (71,624-114,994) | 1,796.0 (1,389.3-2,230.4) |
| 25-44 | 356,100 (355,443-356,775) | 14,970.6 (14,942.9-14,999.0) | 16,236 (10,550-22,188) | 682.5 (443.5-932.8) |
| 45-64 | 133,410 (132,977-133,850) | 10,671.2 (10,636.5-10,706.4) | 7,580 (4,556-10,468) | 606.3 (364.4-837.3) |
| ≥65 | 32,598 (32,381-32,804) | 70,15.2 (6,968.5-7,059.5) | 1,746 (580-3,204) | 375.8 (124.9-689.5) |
| <5 | 233,383 (232,657-234,137) | 12,862.6 (12,822.6-12,904.2) | 29,392 (24,165-34,902) | 1,619.9 (1,331.8-1,923.6) |
| ≥5 | 1,222,180 (1,219,957-1,224,480) | 13,214.0 (13,189.9-13,238.8) | 118,157 (87,310-150,853) | 1,277.5 (944.0-1,631.0) |
| All | 1,455,563 (1,452,614-1,458,617) | 13,156.3 (13,129.7-13,183.9) | 147,549 (111,474-185,755) | 1,333.6 (1,007.6-1,679.0) |
| **Bas-Congo** | | | | |
| <1 | 17,200 (13,654-21,267) | 9,661.6 (7,669.5-11,945.8) | 1,878 (1,274-2,579) | 1,054.9 (715.5-1,448.6) |
| 1-4 | 71,098 (54,636-87,645) | 10,060.3 (7,731-12,401.6) | 9,242 (6,863-12,168) | 1,307.7 (971.1-1,721.8) |
| 5-24 | 264,867 (207,690-326,478) | 10,535.8 (8,261.4-12,986.5) | 35,033 (24,807-46,987) | 1,393.5 (986.7-1,869.1) |
| 25-44 | 134,728 (105,917-170,031) | 11,615.6 (9,131.7-14,659.3) | 6,143 (3,704-9,012) | 529.6 (319.4-777.0) |
| 45-64 | 50,475 (39,729-63,222) | 8,279.8 (6,517.1-10,370.7) | 2,868 (1,668-4,293) | 470.4 (273.6-704.2) |
| ≥65 | 12,333 (9,808-15,237) | 5,443.1 (4,328.5-6,724.9) | 661 (201-1,278) | 291.6 (88.5-563.8) |
| <5 | 88,298 (68,290-108,912) | 9,980.0 (7,718.5-12,309.9) | 11,120 (8,137-14,747) | 1,256.9 (919.6-1,666.8) |
| ≥5 | 462,403 (363,144-574,968) | 10,252.7 (8,051.9-12,748.6) | 44,705 (30,379-61,569) | 991.2 (673.6-1,365.1) |
| All | 550,701 (431,433-683,879) | 10,208.0 (7,997.2-12,676.6) | 55,825 (38,516-76,316) | 1,034.8 (713.9-1,414.6) |
| **Bandundu** | | | | |
| <1 | 30,608 (24,082-38,071) | 10,056.5 (7,912.2-12,508.5) | 3,342 (2,259-4,667) | 1,098.0 (742.2-1,533.4) |
| 1-4 | 126,520 (96,671-156,996) | 10,471.5 (8,001.0-12,993.9) | 16,446 (12,161-21,313) | 1,361.2 (1,006.5-1,764.0) |
| 5-24 | 471,333 (371,357-586,258) | 10,966.4 (8,640.3-13,640.3) | 62,341 (44,156-86,511) | 1,450.5 (1,027.3-2,012.8) |
| 25-44 | 239,749 (187,749-291,178) | 12,090.4 (9,468.1-14,683.8) | 10,931 (6,664-16,197) | 551.2 (336.0-816.8) |
| 45-64 | 89,820 (70,532-111,026) | 8,618.2 (6,767.5-10,652.9) | 5,103 (2,989-7,536) | 489.7 (286.8-723.1) |
| ≥65 | 21,947 (17,183-27,312) | 5,665.6 (4,435.7-7,050.5) | 1,176 (350-2,252) | 303.5 (90.2-581.3) |
| <5 | 157,128 (120,752-195,067) | 10,388.0 (7,983.1-12,896.2) | 19,788 (14,419-25,980) | 1,308.2 (953.3-1,717.6) |
| ≥5 | 822,849 (646,821-1,015,773) | 10,671.7 (8,388.8-13,173.8) | 79,551 (54,158-112,495) | 1,031.7 (702.4-1,459) |
| All | 979,977 (767,573-1210840) | 10,625.2 (8,322.3-13,128.3) | 99,339 (68,577-138,475) | 1,077.1 (743.5-1,501.4) |
| **Équateur** | | | | |
| <1 | 27,737 (22,548-33,269) | 10,056.5 (8,175-12,062.1) | 3,028 (2,083-4,127) | 1,098.0 (755.1-1,496.4) |
| 1-4 | 114,652 (92,637-141,035) | 10,471.5 (8,460.7-12,881.0) | 14,904 (11,318-19,244) | 1,361.2 (1,033.7-1,757.6) |
| 5-24 | 427,123 (350,860-533,372) | 10,966.4 (9,008.3-13,694.3) | 56,493 (40,639-76,078) | 1,450.5 (1,043.4-1,953.3) |
| 25-44 | 217,261 (176,470-269,295) | 12,090.4 (9,820.4-14,986.0) | 9,906 (6,113-14,131) | 551.2 (340.2-786.3) |
| 45-64 | 81,395 (66,524-100,520) | 8,618.2 (7,043.6-10,643.2) | 4,625 (2,652-6,925) | 489.7 (280.8-733.3) |
| ≥65 | 19,888 (16,091-24,218) | 5,665.6 (4,583.9-6,899.0) | 1,065 (324-2,007) | 303.5 (92.3-571.5) |
| <5 | 142,389 (115,185-174,304) | 10,387.9 (8,403.2-12,716.2) | 17,932 (13,401-23,371) | 1,308.2 (977.6-1,705.0) |
| ≥5 | 745,667 (609,944-927,405) | 10,671.7 (8,729.3-13,272.7) | 72,089 (49,727-99,140) | 1,031.7 (711.7-1,418.9) |
| All | 888,056 (725,129-1,101,709) | 10,625.2 (8,675.8-13,181.4) | 90,021 (63,128-122,511) | 1,077.1 (755.3-1,465.8) |
| **Orientale** | | | | |
| <1 | 25,369 (21,594-29,888) | 8,740.2 (7,439.6-10,297.3) | 2,770 (1,954-3,690) | 954.3 (672.9-1,271.1) |
| 1-4 | 104,861 (89,572-124,928) | 9,100.9 (7,773.9-10,842.4) | 13,631 (10,797-17,107) | 1,183.0 (937.1-1,484.7) |
| 5-24 | 390,646 (333,775-461,456) | 9,531 (8,143.5-11,258.6) | 51,669 (38,238-67,314) | 1,260.6 (933.0-1,642.3) |
| 25-44 | 198,707 (169,488-234,448) | 10,507.9 (8,962.7-12,397.9) | 9,060 (5,748-12,815) | 479.1 (304.0-677.6) |
| 45-64 | 74,444 (63,545-87,941) | 7,490.1 (6,393.5-8,848.2) | 4,230 (2,513-6,113) | 425.6 (252.8-615.0) |
| ≥65 | 18,190 (15,414-21,527) | 4,924.0 (4,172.8-5,827.5) | 974 (299-1,872) | 263.8 (81.1-506.6) |
| <5 | 130,230 (111,165-154,816) | 9,028.3 (7,706.6-10,732.7) | 16,401 (12,750-20,796) | 1,137.0 (883.9-1,441.7) |
| ≥5 | 681,987 (582,221-805,372) | 9,274.9 (7,918.1-10,952.9) | 65,933 (46,797-88,112) | 896.7 (636.4-1,198.3) |
| All | 812,217 (693,386-960,188) | 9,234.5 (7,883.4-10,916.8) | 82,334 (59,547-108,908) | 936.1 (677-1,238.2) |
| **Nord-Kivu** | | | | |
| <1 | 16,747 (12,676-21,434) | 7,871.5 (5,958.3-10,074.3) | 1,828 (1,225-2,637) | 859.4 (575.8-1,239.4) |
| 1-4 | 69,222 (53,270-87,762) | 8,196.3 (6,307.3-10,391.4) | 8,998 (6,567-11,957) | 1,065.4 (777.5-1,415.8) |
| 5-24 | 257,878 (19,4487-330,355) | 8,583.6 (6,473.6-10,996.0) | 34,108 (23,317-46,750) | 1,135.3 (776.1-1,556.1) |
| 25-44 | 131,173 (99,738-163,858) | 9,463.4 (7,195.5-11,821.5) | 5,981 (3,536-8,668) | 431.5 (255.0-625.3) |
| 45-64 | 49,143 (37,687-63,944) | 6,745.6 (5,173.0-8,777.3) | 2,792 (1,628-4,190) | 383.3 (223.5-575.2) |
| ≥65 | 12,008 (9,188-15,209) | 4,434.6 (3,393.0-5,617.0) | 643 (186-1,252) | 237.6 (68.4-462.2) |
| <5 | 85,969 (65,946-109,196) | 8,130.9 (6,237.1-10,327.7) | 10,826 (7,792-14,594) | 1,023.9 (736.9-1,380.2) |
| ≥5 | 450,202 (341,098-573,366) | 8,353.0 (6,328.7-10,638.2) | 43,524 (28,666-60,860) | 807.5 (531.9-1,129.2) |
| All | 536,171 (407,044-682,561) | 8,316.6 (6,313.7-10,587.3) | 54,350 (36,457-75,453) | 843.0 (565.5-1,170.4) |
| **Sud-Kivu** | | | | |
| <1 | 20,548 (15,678-25,907) | 11,135.9 (8,496.4-14,039.9) | 2,243 (1,489-3,132) | 1,215.8 (806.9-1,696.9) |
| 1-4 | 84,936 (66,477-106,554) | 11,595.4 (9,075.4-14,546.7) | 11,041 (8,300-14,671) | 1,507.3 (1,133-2,002.8) |
| 5-24 | 316,417 (242,628-400,621) | 12,143.4 (9,311.5-15,375.0) | 41,851 (29,092-57,842) | 1,606.1 (1,116.5-2,219.9) |
| 25-44 | 160,949 (123,954-200,511) | 13,388.1 (10,310.7-16,678.8) | 7,338 (4,403-10,873) | 610.4 (366.3-904.4) |
| 45-64 | 60,298 (47,127-76,424) | 9,543.2 (7,458.7-12,095.3) | 3,426 (1,896-5,175) | 542.2 (300.1-819) |
| ≥65 | 14,733 (11,347-18,820) | 6,273.7 (4,831.8-8,013.6) | 789 (233-1,582) | 336.1 (98.9-673.5) |
| <5 | 105,484 (82,155-132,461) | 11,503.0 (8,958.9-14,444.8) | 13,284 (9,789-17,802) | 1,448.6 (1,067.4-1,941.3) |
| ≥5 | 552,397 (425,055-696,375) | 11,817.1 (9,093.0-14,897.2) | 53,404 (35,623-75,470) | 1,142.4 (762.1-1,614.5) |
| All | 657,881 (507,210-828,836) | 11,765.6 (9,071.0-14,823.0) | 66,688 (45,411-93,272) | 1,192.7 (812.1-1,668.1) |
| **Maniema** | | | | |
| <1 | 10,703 (9,010-12,919) | 14,347.6 (12,076.9-17,316.7) | 1,169 (840-1,608) | 1,566.5 (1,125.2-2,155.9) |
| 1-4 | 44,242 (37,320-52,813) | 14,939.7 (12,601.9-17,833.6) | 5,751 (4,534-7,275) | 1,942.0 (1,531.1-2,456.6) |
| 5-24 | 164,820 (139,946-195,869) | 15,645.7 (13,284.6-18,593.1) | 21,800 (15,926-28,638) | 2,069.4 (1,511.8-2,718.5) |
| 25-44 | 83,838 (70,428-98,450) | 17,249.4 (14,490.3-20,255.8) | 3,822 (2,384-5,441) | 786.4 (490.4-1,119.3) |
| 45-64 | 31,409 (26,825-37,463) | 12,295.6 (10,501-14,665.4) | 1,785 (1,056-2,630) | 698.6 (413.5-1,029.4) |
| ≥65 | 7,675 (6,440-9,064) | 8,083.1 (6,782.6-9,546.1) | 411 (120-780) | 433 (125.4-821.0) |
| <5 | 54,945 (46,329-65,731) | 14,820.3 (12,496.3-17,729.6) | 6,920 (5,374-8,883) | 1,866.5 (1,449.4-2,396.0) |
| ≥5 | 287,742 (243,639-340,845) | 15,225.4 (12,891.8-18,035.3) | 27,818 (19,485-37,487) | 1,471.9 (1,031.0-1,983.6) |
| All | 342,687 (289,968-406,576) | 15,159 (12,826.9-17,985.2) | 34,738 (24,859-46,370) | 1,536.7 (1,099.6-2,051.2) |
| **Katanga** | | | | |
| <1 | 58,242 (51,517-66,659) | 13,873.8 (12,271.8-15,878.8) | 6,359 (4,515-8,403) | 1,514.8 (1,075.6-2,001.6) |
| 1-4 | 240,742 (213,234-275,612) | 14,446.3 (12,795.6-16,538.7) | 31,294 (25,293-38,443) | 1,877.9 (1,517.8-2,306.8) |
| 5-24 | 896,854 (789,325-1,031,468) | 15,129.0 (13,315.1-17,399.8) | 118,622 (89,930-153,230) | 2,001.0 (1,517.0-2,584.8) |
| 25-44 | 456,196 (400,543-517,615) | 16,679.7 (14,644.8-189,25.3) | 20,799 (12,973-29,262) | 760.5 (474.3-1,069.9) |
| 45-64 | 170,910 (149,386-196,307) | 11,889.5 (10,392.2-13,656.2) | 9,711 (5,925-13,805) | 675.5 (412.2-960.3) |
| ≥65 | 41,761 (36,875-48,504) | 7,816.1 (6,901.7-9,078.2) | 2,237 (707-4,222) | 418.7 (132.3-790.2) |
| <5 | 298,984 (264,751-342,271) | 14,331.1 (12,690.2-16,405.9) | 37,653 (29,808-46,845) | 1,804.8 (1,428.8-2,245.4) |
| ≥5 | 1,565,721 (1,376,129-1,793,893) | 14,722.5 (12,939.8-16,868) | 151,369 (109,535-200,518) | 1,423.3 (1,030.0-1,885.5) |
| All | 1,864,705 (1,640,879-2,136,163) | 14,658.3 (12,898.9-16,792.3) | 189,022 (139,343-247,363) | 1,485.9 (1,095.4-1,944.5) |
| **Kasaï Oriental** | | | | |
| <1 | 36,017 (29,921-43,159) | 14,663.5 (12,181.7-17,571.2) | 3,932 (2,742-5,379) | 1,601.0 (1,116.4-2,189.8) |
| 1-4 | 148,877 (121,805-175,817) | 15,268.6 (12,492.1-18,031.4) | 19,353 (14,900-24,643) | 1,984.8 (1,528.1-2,527.4) |
| 5-24 | 554,624 (464,229-660,744) | 15,990.2 (13,384.1-19,049.8) | 73,357 (53,808-96,308) | 2,114.9 (1,551.3-2,776.6) |
| 25-44 | 282,116 (234,856-334,115) | 17,629.2 (14,675.9-20,878.6) | 12,862 (7,893-18,289) | 803.8 (493.2-1,142.8) |
| 45-64 | 105,692 (87,804-124,852) | 12,566.3 (10,439.4-14,844.3) | 6,005 (3,592-8,709) | 714.0 (427-1,035.4) |
| ≥65 | 25,825 (21,119-31,114) | 8,261.1 (6,755.5-9,952.9) | 1,383 (417-2,686) | 442.6 (133.4-859.3) |
| <5 | 184,894 (151,726-218,976) | 15,146.8 (12,429.6-17,938.8) | 23,285 (17,642-30,022) | 1,907.5 (1,445.2-2,459.4) |
| ≥5 | 968,257 (808,007-1,150,824) | 15,560.6 (12,985.3-18,494.6) | 93,607 (65,709-125,992) | 1,504.3 (1,056.0-2,024.8) |
| All | 1,153,151 (959,733-1,369,800) | 15,492.8 (12,894.2-18,403.5) | 116,892 (83,350-156,013) | 1,570.5 (1,119.8-2,096.1) |
| **Kasaï Occidental** | | | | |
| <1 | 20,476 (17,341-23,939) | 10,451.4 (8,851.0-12,219.1) | 2,236 (1,575-2,944) | 1,141.1 (804.0-1,502.7) |
| 1-4 | 84,638 (72,728-99,910) | 10,882.7 (9,351.3-12,846.3) | 11,002 (8,715-13,875) | 1,414.6 (1,120.6-1,784) |
| 5-24 | 315,307 (271,950-369,597) | 11,397.0 (9,829.8-13,359.3) | 41,704 (31,417-53,891) | 1,507.4 (1,135.6-1,947.9) |
| 25-44 | 160,385 (137,174-187,224) | 12,565.1 (10,746.7-14,667.8) | 7,312 (4,682-10,375) | 572.9 (366.9-812.8) |
| 45-64 | 60,087 (51,478-71,338) | 8,956.6 (7,673.3-10,633.7) | 3,414 (2,043-5,016) | 508.9 (304.5-747.6) |
| ≥65 | 14,682 (12,643-17,166) | 5,888.0 (5,070.3-6,884.3) | 787 (244-1,517) | 315.4 (97.7-608.2) |
| <5 | 105,114 (90,069-123,849) | 10,795.9 (9,250.6-12,720.1) | 13,238 (10,290-16,819) | 1,359.6 (1,056.9-1,727.4) |
| ≥5 | 550,461 (473,244-645,324) | 11,090.8 (9,535.0-13,002.1) | 53,217 (38,385-70,798) | 1,072.2 (773.4-1,426.4) |
| All | 655,575 (563,312-769,172) | 11,042.4 (9,488.4-12,955.8) | 66,455 (48,675-87,616) | 1,119.4 (819.9-1,475.8) |

Abbreviations: ILI: influenza-like illness; CI: confidence intervals.

^a^ Rates expressed per 100,000 population.

**Table S3: Estimated mean annual numbers and rates of severe acute respiratory illness and influenza-associated severe acute respiratory illness hospitalizations by province and age group, Democratic Republic of Congo, 2013-2015**

| **Age group (in years)** | **SARI hospitalizations** | | **Influenza-associated SARI hospitalizations** | |
| --- | --- | --- | --- | --- |
|  | **Number (95% CI)** | **Rate (95% CI)^a^** | **Number (95% CI)** | **Rate (95% CI) ^a^** |
| **Kinshasa** | | | | |
| <1 | 22,892 (22,740-23,058) | 6,270.2 (6,228.4-6,315.5) | 1,881 (1,514-2,234) | 515.1 (414.5-611.9) |
| 1-4 | 13,964 (13,836-14,100) | 963.5 (954.7-972.8) | 1,554 (1,262-1,901) | 107.2 (87.1-131.2) |
| 5-24 | 19,671 (19,507-19,825) | 381.5 (378.4-384.5) | 570 (189-1,050) | 11.1 (3.7-20.4) |
| 25-44 | 17,792 (17,647-17,943) | 748 (741.9-754.3) | 571 (164-1,057) | 24.0 (6.9-44.4) |
| 45-64 | 11,311 (11,195-11,432) | 904.7 (895.4-914.4) | 654 (294-1,049) | 52.3 (23.5-83.9) |
| ≥65 | 7,774 (7,682-7,873) | 1,673.1 (1,653.1-1,694.3) | 375 (93-751) | 80.6 (20.1-161.4) |
| <5 | 36,856 (36,576-37,158) | 2,031.3 (2,015.8-2,047.9) | 3,435 (2,776-4,135) | 189.3 (153.0-227.9) |
| ≥5 | 56,548 (56,030-57,072) | 611.4 (605.8-617.1) | 2,170 (739-3,906) | 23.5 (8.0-42.2) |
| All | 93,404 (92,606-94,230) | 844.2 (837-851.7) | 5,605 (3,515-8,041) | 50.7 (31.8-72.7) |
| **Bas-Congo** | | | | |
| <1 | 9,781 (7,652-12,261) | 5,494.1 (4,298.1-6,887.0) | 804 (580-1,050) | 451.4 (325.6-589.7) |
| 1-4 | 5,722 (4,429-7,129) | 809.7 (626.6-1,008.7) | 637 (464-856) | 90.1 (65.7-121.1) |
| 5-24 | 7,040 (5,501-8,744) | 280.1 (218.8-347.8) | 204 (57-383) | 8.1 (2.3-15.2) |
| 25-44 | 6,368 (4,992-8,032) | 549.0 (430.3-692.4) | 204 (60-384) | 17.6 (5.1-33.1) |
| 45-64 | 4,048 (3,128-5,031) | 664.1 (513.2-825.2) | 234 (102-397) | 38.4 (16.6-65.1) |
| ≥65 | 2,783 (2,204-3,439) | 1,228.0 (972.7-1,517.7) | 134 (29-271) | 59.2 (13.0-119.5) |
| <5 | 15,503 (12,081-19,390) | 1,752.2 (1,365.4-2,191.5) | 1,441 (1,044-1,906) | 162.9 (118.0-215.4) |
| ≥5 | 20,239 (15,825-25,245) | 448.8 (350.9-559.7) | 776 (247-1,434) | 17.2 (5.5-31.8) |
| All | 35,742 (27,905-44,634) | 662.5 (517.3-827.4) | 2,217 (1,291-3,340) | 41.1 (23.9-61.9) |
| **Bandundu** | | | | |
| <1 | 16,616 (12,927-20,817) | 5,459.4 (4,247.3-6,839.4) | 1,365 (983-1,833) | 448.5 (322.9-602.2) |
| 1-4 | 9,733 (7,376-12,297) | 805.5 (610.4-1,017.7) | 1,083 (782-1,466) | 89.6 (64.7-121.3) |
| 5-24 | 12,616 (9,998-15,642) | 293.5 (232.6-364) | 366 (106-716) | 8.5 (2.5-16.7) |
| 25-44 | 11,411 (8,914-14,038) | 575.4 (449.5-707.9) | 366 (103-697) | 18.5 (5.2-35.1) |
| 45-64 | 7,254 (5,652-8,987) | 696.0 (542.3-862.3) | 419 (182-698) | 40.2 (17.5-66.9) |
| ≥65 | 4,986 (3,925-6,195) | 1,287.2 (1,013.3-1,599.1) | 240 (53-507) | 62.0 (13.6-130.9) |
| <5 | 26,349 (20,303-33,114) | 1,742.0 (1,342.3-2,189.2) | 2,448 (1,764-3,299) | 161.8 (116.6-218.1) |
| ≥5 | 36,267 (28,488-44,861) | 470.4 (369.5-581.8) | 1,391 (444-2,617) | 18.0 (5.8-33.9) |
| All | 62,616 (48,791-77,974) | 678.9 (529.0-845.4) | 3,839 (2,208-5,916) | 41.6 (23.9-64.1) |
| **Équateur** | | | | |
| <1 | 15,227 (12,415-18,453) | 5,520.6 (4,501.1-6,690.4) | 1,251 (919-1,623) | 453.5 (333.1-588.3) |
| 1-4 | 9,007 (7,171-11,048) | 822.6 (654.9-1,009.0) | 1,002 (742-1,327) | 91.5 (67.7-121.1) |
| 5-24 | 11,709 (9,527-14,629) | 300.6 (244.6-375.6) | 339 (101-657) | 8.7 (2.6-16.9) |
| 25-44 | 10,590 (8,626-13,159) | 589.3 (480.0-732.3) | 340 (98-632) | 18.9 (5.4-35.2) |
| 45-64 | 6,732 (5,470-8,318) | 712.8 (579.2-880.8) | 389 (165-637) | 41.2 (17.4-67.4) |
| ≥65 | 4,627 (3,739-5,608) | 1,318.2 (1,065.1-1,597.6) | 223 (51-466) | 63.5 (14.6-132.7) |
| <5 | 24,234 (19,586-29,501) | 1,768.0 (1,428.9-2,152.2) | 2,253 (1,660-2,950) | 164.4 (121.1-215.2) |
| ≥5 | 33,658 (27,362-41,714) | 481.7 (391.6-597) | 1,291 (414-2,391) | 18.5 (5.9-34.2) |
| All | 57,892 (46,947-71,214) | 692.7 (561.7-852) | 3,544 (2,074-5,341) | 42.4 (24.8-63.9) |
| **Orientale** | | | | |
| <1 | 13,744 (11,558-16,238) | 4,735.1 (3,982.1-5,594.3) | 1,129 (865-1,423) | 389.0 (298.0-490.0) |
| 1-4 | 8,564 (7,227-10,291) | 743.2 (627.2-893.1) | 953 (715-1,261) | 82.7 (62.0-109.5) |
| 5-24 | 11,730 (10,024-13,895) | 286.2 (244.6-339.0) | 340 (105-658) | 8.3 (2.6-16.0) |
| 25-44 | 10,609 (9,103-12,480) | 561.0 (481.3-659.9) | 341 (95-625) | 18.0 (5.0-33.0) |
| 45-64 | 6,744 (5,709-8,020) | 678.6 (574.3-806.8) | 390 (180-663) | 39.2 (18.1-66.7) |
| ≥65 | 4,636 (3,940-5,519) | 1,254.9 (1,066.6-1,493.8) | 223 (51-458) | 60.5 (13.6-124.1) |
| <5 | 22,308 (18,785-26,529) | 1,546.5 (1,302.3-1,839.1) | 2,082 (1,580-2,684) | 144.3 (109.5-186.0) |
| ≥5 | 33,719 (28,776-39,912) | 458.6 (391.3-542.8) | 1,294 (430-2,404) | 17.6 (5.8-32.7) |
| All | 56,027 (47,560-66,441) | 637.0 (540.7-755.4) | 3,376 (2,010-5,087) | 38.4 (22.8-57.8) |
| **Nord-Kivu** | | | | |
| <1 | 9,599 (7,285-12,411) | 4.511.8 (3.424.0-5,833.5) | 789 (574-1,080) | 370.7 (269.7-507.5) |
| 1-4 | 5,736 (4,413-7,335) | 679.2 (522.5-868.5) | 638 (443-865) | 75.6 (52.4-102.5) |
| 5-24 | 7,526 (5,703-9,652) | 250.5 (189.8-321.3) | 218 (62-427) | 7.3 (2.0-14.2) |
| 25-44 | 6,807 (5,176-8,553) | 491.1 (373.4-617.1) | 219 (68-425) | 15.8 (4.9-30.6) |
| 45-64 | 4,327 (3,320-5,644) | 594.0 (455.6-774.7) | 250 (105-426) | 34.3 (14.4-58.5) |
| ≥65 | 2,974 (2,266-3,764) | 1,098.4 (837.0-1,390.0) | 143 (31-300) | 52.9 (11.2-110.7) |
| <5 | 15,335 (11,698-19,746) | 1,450.4 (1,106.3-1,867.5) | 1,427 (1,016-1,945) | 135.0 (96.1-184.0) |
| ≥5 | 21,634 (16,464-27,612) | 401.4 (305.5-512.3) | 830 (265-1,577) | 15.4 (4.9-29.3) |
| All | 36,969 (28,161-47,358) | 573.4 (436.8-734.6) | 2,257 (1,281-3,522) | 35.0 (19.9-54.6) |
| **Sud-Kivu** | | | | |
| <1 | 11,268 (8,392-14,349) | 6,106.4 (4,548.1-7,776.2) | 926 (666-1,233) | 501.7 (361-667.8) |
| 1-4 | 6,623 (5,128-8,469) | 904.2 (700.0-1,156.2) | 737 (525-1,016) | 100.6 (71.7-138.6) |
| 5-24 | 8,240 (6,247-10,486) | 316.2 (239.7-402.4) | 239 (69-476) | 9.2 (2.6-18.3) |
| 25-44 | 7,453 (5,759-9,381) | 619.9 (479.0-780.3) | 239 (69-444) | 19.9 (5.7-37.0) |
| 45-64 | 4,738 (3,687-5,987) | 749.9 (583.4-947.5) | 274 (115-465) | 43.3 (18.1-73.6) |
| ≥65 | 3,257 (2,528-4,139) | 1,386.7 (1,076.2-1,762.3) | 157 (35-330) | 66.8 (14.9-140.3) |
| <5 | 17,891 (13,520-22,818) | 1,951.0 (1,474.3-2,488.2) | 1,663 (1,191-2,248) | 181.3 (129.9-245.1) |
| ≥5 | 23,688 (18,219-29,992) | 506.7 (389.7-641.6) | 909 (287-1,714) | 19.4 (6.1-36.7) |
| All | 41,579 (31,739-52,810) | 743.6 (567.6-944.4) | 2,572 (1,478-3,962) | 46.0 (26.4-70.9) |
| **Maniema** | | | | |
| <1 | 6,354 (5,283-7,785) | 8,517.1 (7,080.8-10,435.0) | 522 (389-684) | 699.7 (521.5-916.2) |
| 1-4 | 4,136 (3,472-4,985) | 1,396.7 (1,172.4-1.683.2) | 460 (345-603) | 155.4 (116.4-203.6) |
| 5-24 | 5,094 (4,317-6,090) | 483.6 (409.8-578.1) | 148 (45-280) | 14.0 (4.3-26.6) |
| 25-44 | 4,608 (3,847-5,440) | 948.0 (791.4-1.119.1) | 148 (43-274) | 30.4 (8.8-56.5) |
| 45-64 | 2,929 (2,493-3,498) | 1,146.7 (975.8-1.369.1) | 169 (74-281) | 66.3 (29.0-110.1) |
| ≥65 | 2,013 (1,678-2,395) | 2,120.5 (1,766.8-2.522.5) | 97 (22-205) | 102.2 (23.5-215.5) |
| <5 | 10,490 (8,755-12,770) | 2,829.5 (2,361.4-3.444.3) | 982 (734-1.287) | 264.9 (198.0-347.0) |
| ≥5 | 14,644 (12,333-17,422) | 774.9 (652.6-921.9) | 562 (183-1.040) | 29.7 (9.7-55.0) |
| All | 25,134 (21,088-30,192) | 1,111.8 (932.8-1,335.5) | 1,544 (917-2,326) | 68.3 (40.6-102.9) |
| **Katanga** | | | | |
| <1 | 32,195 (28,187-37,556) | 7,669.3 (6,714.3-8,946.0) | 2,645 (2,022-3,370) | 630.1 (481.6-802.7) |
| 1-4 | 19,580 (17,126-22,701) | 1,174.9 (1,027.7-1,362.3) | 2,179 (1,699-2,826) | 130.7 (101.9-169.6) |
| 5-24 | 26,354 (23,219-30,369) | 444.6 (391.7-512.3) | 764 (235-1,461) | 12.9 (4.0-24.6) |
| 25-44 | 23,837 (20,821-27,165) | 871.5 (761.3-993.2) | 765 (217-1,413) | 28.0 (7.9-51.7) |
| 45-64 | 15,154 (13,185-17,452) | 1,054.2 (917.2-1,214.1) | 876 (389-1,453) | 60.9 (27.0-101.1) |
| ≥65 | 10,416 (9,158-12,217) | 1,949.5 (1,714.0-2,286.5) | 502 (118-1,027) | 94.0 (22.0-192.1) |
| <5 | 51,775 (45,313-60,257) | 2,481.7 (2,171.9-2,888.2) | 4,824 (3,721-6,195) | 231.2 (178.4-296.9) |
| ≥5 | 75,761 (66,382-87,202) | 712.4 (624.2-820.0) | 2,907 (959-5,352) | 27.3 (9.0-50.3) |
| All | 127,536 (111,694-147,459) | 1,002.6 (878.0-1,159.2) | 7,731 (4,680-11,547) | 60.8 (36.8-90.8) |
| **Kasaï Oriental** | | | | |
| <1 | 20,577 (16,820-25,035) | 8,377.6 (6,847.9-10,192.1) | 1,690 (1,258-2,158) | 688.2 (512.1-878.3) |
| 1-4 | 12,625 (10,235-15,008) | 1,294.8 (1,049.6-1,539.1) | 1,405 (1,032-1,826) | 144.1 (105.9-187.3) |
| 5-24 | 16,220 (13,620-19,304) | 467.6 (392.7-556.6) | 470 (145-882) | 13.6 (4.1-25.4) |
| 25-44 | 14,671 (12,226-17,338) | 916.8 (763.9-1,083.4) | 471 (140-895) | 29.4 (8.8-55.9) |
| 45-64 | 9,326 (7,750-10,999) | 1,108.9 (921.4-1,307.8) | 539 (230-894) | 64.1 (27.3-106.3) |
| ≥65 | 6,410 (5,251-7,675) | 2,050.6 (1,679.6-2,454.9) | 309 (70-650) | 98.8 (22.3-207.8) |
| <5 | 33,202 (27,055-40,042) | 2,720.0 (2,216.4-3,280.3) | 3,095 (2,290-3,984) | 253.5 (187.6-326.3) |
| ≥5 | 46,627 (38,847-55,315) | 749.3 (624.3-889) | 1,789 (584-3,320) | 28.8 (9.4-53.3) |
| All | 79,829 (65,902-95,357) | 1,072.5 (885.4-1,281.1) | 4,884 (2,873-7,303) | 65.6 (38.6-98.1) |
| **Kasaï Occidental** | | | | |
| <1 | 12,165 (10,287-14,447) | 6,209.5 (5,250.5-7,373.7) | 999 (772-1,277) | 510.1 (394.0-651.6) |
| 1-4 | 7,213 (6,097-8,652) | 927.5 (783.8-1,112.3) | 803 (600-1,037) | 103.2 (77.1-133.3) |
| 5-24 | 8,977 (7,648-10,525) | 324.5 (276.5-380.5) | 260 (77-505) | 9.4 (2.8-18.2) |
| 25-44 | 8,119 (6,891-9,486) | 636.1 (539.9-743.2) | 261 (75-489) | 20.4 (5.9-38.3) |
| 45-64 | 5,161 (4,397-6,168) | 769.4 (655.4-919.3) | 298 (128-487) | 44.5 (19.1-72.6) |
| ≥65 | 3,548 (3,044-4,148) | 1,422.8 (1,220.6-1,663.5) | 171 (38-337) | 68.6 (15.4-135.3) |
| <5 | 19,378 (16,383-23,098) | 1,990.2 (1,682.6-2,372.3) | 1,802 (1,372-2,313) | 185.1 (140.9-237.6) |
| ≥5 | 25,805 (21,980-30,327) | 519.9 (442.8-611.0) | 990 (318-1817) | 19.9 (6.4-36.6) |
| All | 45,183 (38,363-53,425) | 761.1 (646.2-899.9) | 2,792 (1,690-4,130) | 47.0 (28.5-69.6) |

Abbreviations: SARI: severe acute respiratory illness; CI: confidence intervals.

^a^ Rates expressed per 100,000 population.
